# Diffusivity in the core of chronic multiple sclerosis lesions

**DOI:** 10.1371/journal.pone.0194142

**Published:** 2018-04-25

**Authors:** Alexander Klistorner, Chenyu Wang, Con Yiannikas, John Parratt, Joshua Barton, Yuyi You, Stuart L. Graham, Michael H. Barnett

**Affiliations:** 1 Save Sight Institute, Sydney Medical School, University of Sydney, Sydney, Australia; 2 Faculty of Medicine and Health Sciences, Macquarie University, Sydney, New South Wales, Australia; 3 Sydney Neuroimaging Analysis Centre, Sydney, New South Wales, Australia; 4 Brain and Mind Centre, University of Sydney, Sydney, New South Wales, Australia; 5 Royal North Shore Hospital, Sydney, New South Wales, Australia; McLean Hospital, UNITED STATES

## Abstract

**Background:**

Diffusion tensor imaging (DTI) has been suggested as a potential biomarker of disease progression, neurodegeneration and de/remyelination in MS. However, the pathological substrates that underpin alterations in brain diffusivity are not yet fully delineated. We propose that in highly cohesive fiber tracts: 1) a relative increase in parallel (axial) diffusivity (AD) may serve as a measure of increased extra-cellular space (ESC) within the core of chronic MS lesions and, as a result, may provide an estimate of the degree of tissue destruction, and 2) the contribution of the increased extra-cellular water to perpendicular (radial) diffusivity (RD) can be eliminated to provide a more accurate assessment of membranal (myelin) loss.

**Objective:**

The purpose of this study was to isolate the contribution of extra-cellular water and demyelination to observed DTI indices in the core of chronic MS lesions, using the OR as an anatomically cohesive tract.

**Method:**

Pre- and post-gadolinium (Gd) enhanced T1, T2 and DTI images were acquired from 75 consecutive RRMS patients. In addition, 25 age and gender matched normal controls were imaged using an identical MRI protocol (excluding Gd). The optic radiation (OR) was identified in individual patients using probabilistic tractography. The T2 lesions were segmented and intersected with the OR. Average eigenvalues were calculated within the core of OR lesions mask. The proportion of extra-cellular space (ECS) within the lesional core was calculated based on relative increase of AD, which was then used to normalise the perpendicular eigenvalues to eliminate the effect of the expanded ECS. In addition, modelling was implemented to simulate potential effect of various factors on lesional anisotropy.

**Results:**

Of 75 patients, 41 (55%) demonstrated sizable T2 lesion volume within the ORs. All lesional eigenvalues were significantly higher compared to NAWM and controls. There was a strong correlation between AD and RD within the core of OR lesions, which was, however, not seen in OR NAWM of MS patients or normal controls. In addition, lesional anisotropy (FA) was predominantly driven by the perpendicular diffusivity, while in NAWM and in OR of normal controls all eigenvectors contributed to variation in FA.

Estimated volume of ECS component constituted significant proportion of OR lesional volume and correlated significantly with lesional T1 hypointensity.

While perpendicular diffusivity dropped significantly following normalisation, it still remained higher compared with diffusivity in OR NAWM. The “residual” perpendicular diffusivity also showed a substantial reduction of inter-subject variability. Both observed and modelled diffusion data suggested anisotropic nature of water diffusion in ESC. In addition, the simulation procedure offered a possible explanation for the discrepancy in relationship between eigenvalues and anisotropy in lesional tissue and NAWM.

**Conclusion:**

This paper presents a potential technique for more reliably quantifying the effects of neurodegeneration (tissue loss) versus demyelination in OR MS lesions. This may provide a simple and effective way for applying single tract diffusion analysis in MS clinical trials, with particular relevance to pro-remyelinating and neuroprotective therapeutics.

## Introduction

Multiple sclerosis (MS) is a complex disease of the CNS, characterized by inflammation, demyelination, neuro-axonal loss and gliosis[1]. Inflammatory demyelinating lesions are a hallmark of the disease. However, neuro-axonal loss is believed to underpin the progressive disability that characterizes MS.

Conventional magnetic resonance imaging (MRI) supplements clinical assessment and is considered the “gold standard” investigation for MS diagnosis. However, MRI has limited capacity to distinguish between the characteristic pathological features of the disease. Significant expansion of the therapeutic options for MS over the last several years has re-emphasized the critical need for reliable in-vivo markers of disease progression and neurodegeneration. In addition, recent interest in the development of remyelinating therapies has created a demand for reliable *in vivo* surrogate markers of remyelination.

Diffusion tensor imaging (DTI) has been suggested as one potential new biomarker. DTI is sensitive to the microstructural organisation of white matter tracts and provides greater pathological specificity than conventional MRI, helping, therefore, to elucidate disease pathogenesis and monitor therapeutic efficacy[2]. However, the pathological substrates that underpin alterations in brain diffusivity are not yet fully delineated. Post-mortem and animal studies may not be directly comparable or applicable to *in vivo* human pathology, while clinical studies of diffusivity are difficult to validate since histological correlations are not feasible. In addition, the fundamental dissociation between the dimensions of tissue microstructure (10–100 μ) and DTI resolution (typical voxel size—2 mm, which may contain up to 5 million axons [3]) presents a major impediment to understanding the nature of diffusivity alteration in brain disorders and prevent direct (histological) verification of diffusivity measures.

While it is not possible to directly measure water diffusion within the various tissue compartments of a single voxel, some insights into the specificity of diffusion indices can be indirectly deduced from our knowledge of tissue pathology, which is well described in MS. Thus, a single voxel may be thought of as a ‘black box’ with different pathological features of MS treated as an input, and resulting abnormal diffusivity as an output [4]. This approach can, for instance, be applied to the core of chronic MS lesions, the features of which are well characterized and represented by the varying degrees of extra-cellular space (ECS) expansion, demyelination of preserved axons and gliosis [5][6][7][8]. Widening of the ECS (caused largely by tissue destruction and axonal loss) is likely to result in an increase in diffusion of the water molecules in all directions, affecting both parallel and perpendicular diffusivity. Conversely, loss of myelin membranes, particularly in highly cohesive fiber tracts (such as optic radiation) may have a dramatic effect on perpendicular (radial) diffusivity (RD), but is unlikely to significantly affect the diffusivity parallel to axonal fibers (axial diffusivity, AD). Consequently, while at least two major features of chronic MS lesions (demyelination and expanded ECS) may contribute to the increase in RD of a single fiber tract, only the latter is likely to affect the AD.

We hypothesized, therefore, that a relative increase in AD may serve as a measure of increased ESC within chronic MS lesions and, as a result, may provide an estimate of the degree of tissue destruction (at least in highly cohesive fiber tracts). We further speculated that, based on this knowledge the contribution of the increased extra-cellular water to RD can be minimized (or eliminated) with “residual” RD providing a more accurate measure of membranal (myelin) loss.

The specificity of altered diffusion for pathologic changes is limited by the wide spectrum of normal anisotropy indices in the brain [9]. We studied lesions in the optic radiations, highly organized fibre tracts that are a frequent site of MS pathology, to facilitate accurate measurement of relative diffusivity change along axonal bundles[10]. In addition, internal structure of the OR does not contain a significant number of crossing fibers, which can potentially (and sometimes paradoxically) alter diffusivity[11][12]. This point is especially pertinent considering the issues that surround misalignment between corresponding eigenvectors with the underlying tissue structures[13].

The current study represents the first attempt to apply this methodology to investigate diffusivity in the core of chronic MS lesions within a single white matter pathway in patients with RRMS. This task was approached in two ways: firstly, by performing analysis of the clinical data and secondly, by implementing simulation modeling.

## Material and methods

The study was approved by University of Sydney and Macquarie University Human Research Ethics Committees. All procedures followed the tenets of the Declaration of Helsinki and written informed consent was obtained from all participants.

### Subjects

Seventy-five consecutive patients with Relapsing-Remitting MS (RRMS) were enrolled. RRMS was defined according to standard criteria [14]. A history of optic neuritis (ON) in one eye was not an exclusion criteria, however, none of the patients had ON or new visual symptoms 6 months prior to the study. All patients with a history of ON had received steroid therapy as part of their acute ON treatment. A history of ON was based on the patient’s clinical notes and the absence of previous visual symptoms on direct questioning. Patients with any other systemic or ocular disease, in particular those that could potentially affect our measurement parameters were excluded.

In addition, 25 age and gender matched normal controls (age 40.0±9.5, 6M/19F) were imaged using an identical MRI protocol (excluding Gd).

### MRI protocol

The following sequences were acquired using a 3T GE Discovery MR750 scanner (GE Medical Systems, Milwaukee, WI):

1. Pre- and post contrast (gadolinium) Sagittal 3D T1: GE BRAVO sequence, duration 4 min each, FOV 256mm, Slice thickness 1mm, TE 2.7ms, TR 7.2ms, Flip angle 12°, Pixel spacing 1mm. Acquisition Matrix (Freq.× Phase) is 256×256, which results in 1mm isotropic acquisition voxel size. The reconstruction matrix is 256x256.
2. FLAIR CUBE; GE CUBE T2 FLAIR sequence, duration 6 min, FOV 240mm, Slice thickness 1.2mm, Acquisition Matrix (Freq.× Phase) 256×244, TE 163ms, TR 8000ms, Flip angle 90°, Pixel spacing 0.47 mm. The reconstruction matrix is 512x512.
3. Echo-Planar Imaging based diffusion weighted MRI, duration 9 min (64-directions with 2mm isotropic acquisition matrix, TR/TE = 8325/86 ms, b = 1000 s/mm^2^, number of b0s = 2).

### Reconstruction of individual optic radiations

**Fig 1:**
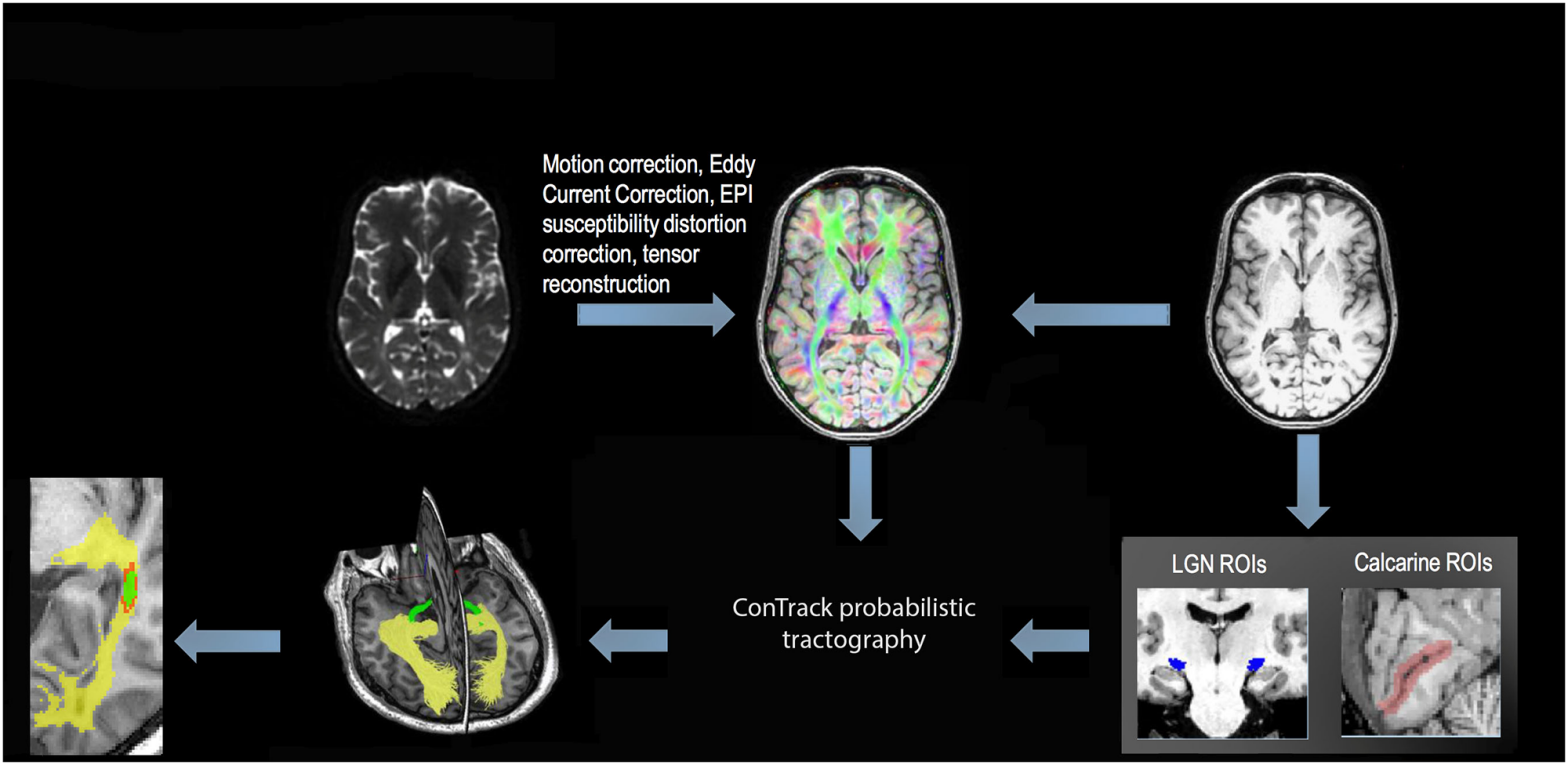
Image analysis pipeline. After motion, Eddy current and EPI distortion corrections, DTI image was coregistered with structural T1 image. ConTrack probabilistic tractography using previously identified LGN and calcarine ROIs was performed. After lesion erosion, whole brain lesion mask was intersected with optic radiations.

Probabilistic tractography was used to reconstruct OR fibers as previously described in detail elsewhere[15] (Fig 1). Briefly, after eddy-current correction and motion compensation, DTI and FLAIR T2 images were co-registered to the high resolution T1 structural image. To reduce the effect of EPI susceptibility distortion, non-linear registration-based correction was used for DTI co-registration. Identification of two regions of interest (ROI), the lateral geniculate nucleus (LGN) and the occipital cortex, facilitated the implementation of probabilistic tractography of the optic radiation (OR). To identify the LGN, which is nearly invisible on structural T1-weighted images, optic tract fibers were followed from the optic chiasm using deterministic tractography (a 10 mm ROI placed on the optic chiasm was used to seed the deterministic algorithm). The position of the LGN was inferred by the termination of optic tract fibers, at which point a circular ROI (diameter 7 mm) was placed. An occipital cortex ROI covering the calcarine sulcus was manually drawn on the high resolution T1 structural image in each hemisphere using the FSL software (www.fmrib.ox.ac.uk). Seeding ROIs were derived for each subject individually. Probabilistic tractography was then employed between the LGN and calcarine ROIs using the ConTrack feature of MrDiffusion software (http://sirl.stanford.edu/software/) and parameters described by Sherbondy et al [16]. Initially, 70000 fibers were collected for OR tractography, of which the 30000 best fibers were selected by a scoring algorithm. OR fibers were then manually cleaned using Quench software (http://sirl.stanford.edu/software/). Meyer’s loop was clearly visible in all OR reconstructions.

### Lesion identification and analysis

Whole brain T2 lesions were identified on the co-registered T2 FLAIR images and semi-automatically segmented using JIM 7 software (Xinapse Systems, Essex, UK) by a trained analyst. To minimize partial volume effect [17] and to exclude lesion edge, the lesions were shrunk by 1 voxel in all directions using the “eroding” function of the JIM software.

The eroded lesion mask was intersected with the OR mask and applied to DTI images to calculate diffusivity in the “core” of OR lesions.

The eroded lesion mask was also applied to pre-contrast 3D-T1-weighted images to quantify lesion hypointensity. In order to reduce inter-subject variability, the lesional hypointensity was normalised by the intensity of neighboring NAWM, which was measured using additional 2 mm ROIs placed in NAWM of both hemispheres in close proximity to the lesions.

Gd enhancing lesions were excluded from the analysis.

Twenty-one patients did not show any visible lesions within OR bilaterally and, therefore, their entire OR was considered as NAWM. Similar approach was applied to normal controls.

### DTI analysis

This study is based on the hypothesis that in highly cohesive fiber tracts such as the OR, an increase of water diffusion along the direction parallel to the main fiber orientation (i.e.AD) is predominantly driven by enlargement of the ECS secondary to the tissue loss. AD, therefore, can be used to indirectly infer the amount of increased water content (and corresponding tissue loss) by computing the increase of AD in lesions compared to NAWM.

Based on this assumption, we interpreted the measured diffusion in a single voxel of white matter as a linear combination of two components: a “ECS” component and “normal tissue” component with no water exchange between the two.

$${AD}^{measrued}=f∙{AD}^{normal\;tissue}+(1-f)∙{AD}^{ECS}$$

Where:

"*f*"—volume fraction of normal tissue

“1 − *f*”—volume fraction of “ECS”

*AD*^*normal tissue*^- parallel water diffusion in OR NAWM (1.33 x10^−3^ mm^2^ s^−1^)

*AD*^*ECS*^—parallel water diffusion in the ECS of OR lesions (see below)

Therefore, the volume fraction of the lesional “ECS” (*f)* was calculated as follow:

$$f=\left({AD}^{measrued}-{AD}^{ECS}\right)/\left({AD}^{normal\;tissue}-{AD}^{ECS}\right)$$

Based on the calculated fraction of the “ECS” component the perpendicular eigenvalues (*λ*_2,3_) were normalised to eliminate the effect of the expanded ECS using the following formula:

$$\lambda_{norm}=\lambda_{measrued}-\alpha ∙(1-f)$$

where:

*α* is the slope of the correlation function between *λ* and *f*

Radial Diffusivity (RD) was calculated as an average between *λ*_2_ and *λ*_3_.

Since the “normal tissue “in the core of chronic MS lesions is represented by fully demyelinated axons and assuming that there is minimal or no effect of demyelination on axial diffusivity, we hypothesised that the “ECS” component primarily reflects the degree of axonal loss. However, tissue destruction in MS lesions is also known to be accompanied by severe gliosis, which can significantly effect on diffusion of water molecules within the tissue. Thus, a recent study by Budde et al [18] demonstrated that, following brain injury, the elongated processes of glial cells display directional cohesiveness that can result in a degree of anisotropy. This may be particularly relevant to diffusion in highly coherent fiber tracts such as the OR. Therefore, to determine if the diffusion of water molecules in extensively damaged areas of MS lesions is similar to the diffusion of free water, we examined AD and RD voxel-based histograms of OR lesions.

### Simulation

We simulated the effects of several factors on eigenvalues and their relationship with anisotropy including:

1. an increase of the ECS
2. membranal loss
3. inter-subject variability

The simulation model assumes that total eigenvalues of the lesional tissue $\left(\lambda_{1,2,3}^{\left(lesion\right)}\right)$ are the linear sum of following eigenvalues:

$$\lambda_{1}^{\left(lesion\right)}=f∙\lambda_{1}^{\left(normal\;tissue\right)}+(1-f)∙\lambda_{1}^{\left(ECS\right)}+\lambda_{1}^{\left(noise\right)}$$

$$\lambda_{2,3}^{\left(lesion\right)}=f∙\lambda_{2,3}^{\left(normal\;tissue\right)}+(1-f)∙\lambda_{2,3}^{\left(ECS\right)}+\lambda_{2,3}^{\left(noise\right)}+\lambda_{2,3}^{\left(membrane\right)}$$

Where:

*λ*^(*normal tissue*)^ is eigenvalue in OR NAWM,

*λ*^(*ECS*)^ is eigenvalue in ECS

*f* is normal tissue volume fraction,

*λ*_2,3_^(membrane)^ is change (reduction) of perpendicular diffusivity caused by membrane (myelin) loss

*λ*^(*noise*)^ is inter-subject noise

An enlargement of the ECS was simulated by increasing the proportion of the “ECS” compartment, i.e. anisotropic diffusivity in OR NAWM was randomly replaced by the diffusivity of water in the ECS. The proportion of replacement was based on the observed distribution of the “ECS” compartment and was determined using the following formula:

=NORMINV(RAND(),Mean("ECS"),SD("ECS"))

Membranal loss was modelled as an increase of perpendicular diffusivity (both *λ*_2_ and *λ*_3_) compared to the NAWM. It was randomly generated based on following formula:

=NORMINV(RAND(),Mean(A),SD(α))

where *A* is mean difference between RD in NAWM and normalised (“residual”) perpendicular diffusivity and *SD*(*α*) is Standard Deviation of “residual” perpendicular diffusivity.

Inter-subject variability for each eigenvalue was established using normal control data and was added to the eigenvalues as a random number based on normal cumulative distribution of diffusivity differences between subjects.

A dataset of one hundred diffusivity combinations was created and analysed.

### Statistical analysis

Statistical analysis was performed using SPSS 22.0 (SPSS, Chicago, IL, USA). Comparisons between groups were made using unpaired Student’s t-test (for two groups) or one-way ANOVA (Tukey post-hoc analysis for multiple groups). Pearson correlation coefficient was used to measure statistical dependence between two numerical variables. P < 0.05 was considered statistically significant. Variability of different parameters was assessed by the coefficient of variation (CV), calculated as standard deviation divided by the mean of the measured values. D’Agostino-Pearson omnibus normality test was used to determine whether data were sampled from Gaussian distributions.

## Results

Seventy-five consecutive RRMS patients (age: 41.6±10.1, disease duration: 4.9±3.6 y, 25M/50F, EDSS score: 1.42±1.38) were enrolled in the study. Seventy patients (93%) were receiving disease-modifying therapy at the time of enrolment (7-beta-interferon 1b, 20-glatiramer acetate, 25-fingolimod, 6-natalizumab, 10-interferon-beta 1a, 2-dimethyl fumarate).

Of 75 patients, 41 (55%) demonstrated sizable (>100 mm^3^) T2 lesion volume within the ORs. The mean volume of the “core” of OR lesions was 796±1033mm^3^.

**Fig 2:**
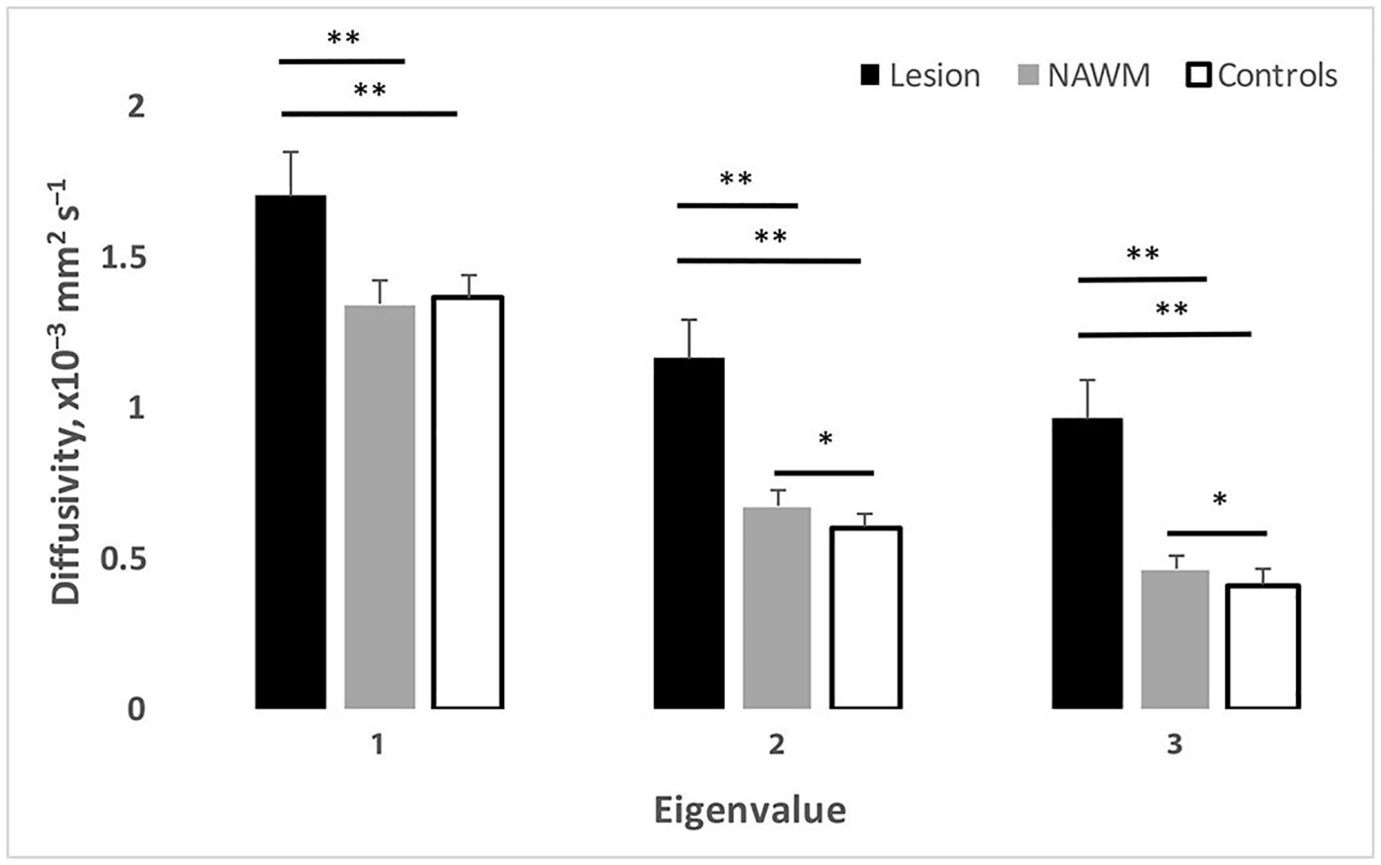
Eigenvalues in OR lesions, OR NAWM and OR of normal controls. * p<0.05, ** p<0.01.

Diffusivity indices within OR lesions and NAWM of MS patients and within control’s ORs are presented in Table 1 and Fig 2. While all lesional eigenvalues were significantly higher compared to NAWM and controls (p<0.0001 for all, one-way ANOVA), AD (λ_1_) did not differ between the latter two groups (p = 0.6, one-way ANOVA). Perpendicular eigenvalues, however, were higher in NAWM compared to normal controls (p = 0.009 and 0.046 for λ_2_ and λ_2_ respectively), resulting in a significant increase of RD in the former (p = 0.019). FA was significantly different between all three group, demonstrating, predictably, the lowest value in lesions and the highest in normal controls (p<0.0001 for both, one-way ANOVA).

**Table 1:** Diffusivity indices in OR lesions, NAWM and normal controls.

	n	AD	RD	FA
Lesion	41	1.7±0.15	1.06±0.13	0.29±0.03
NAWM	21	1.33±0.08	0.57±0.05	0.50±0.05
Controls	25	1.36±0.08	0.50±0.04	0.57±0.05

**Fig 3:**
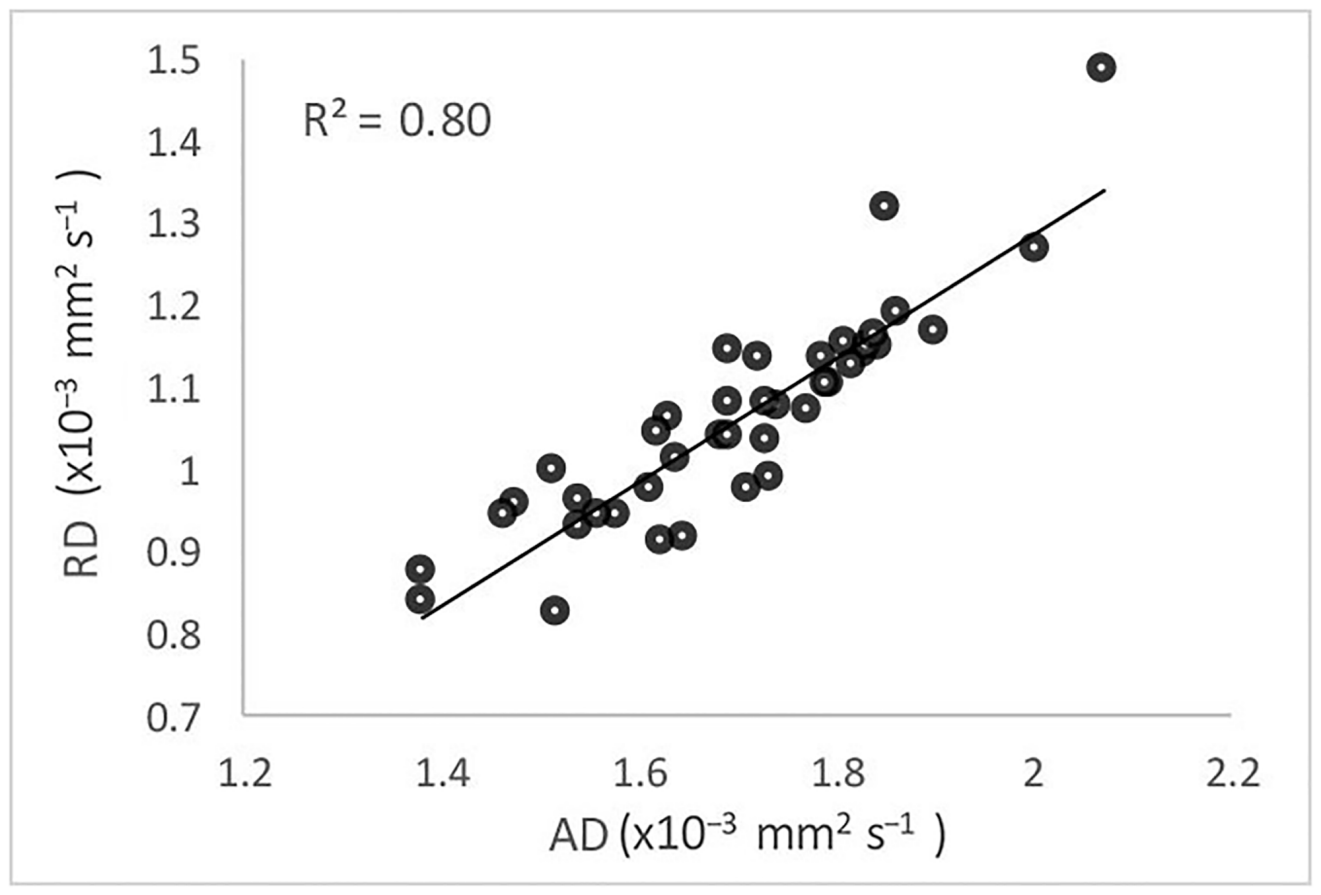
Correlation between AD and RD in OR lesions.

There was a strong correlation between AD and RD within OR lesions (r = 0.90) (Fig 3). This correlation, however, was not seen in OR NAWM in MS patients or in the OR of normal controls (r^2^ = 0.005 and 0.06 respectively).

**Fig 4:**
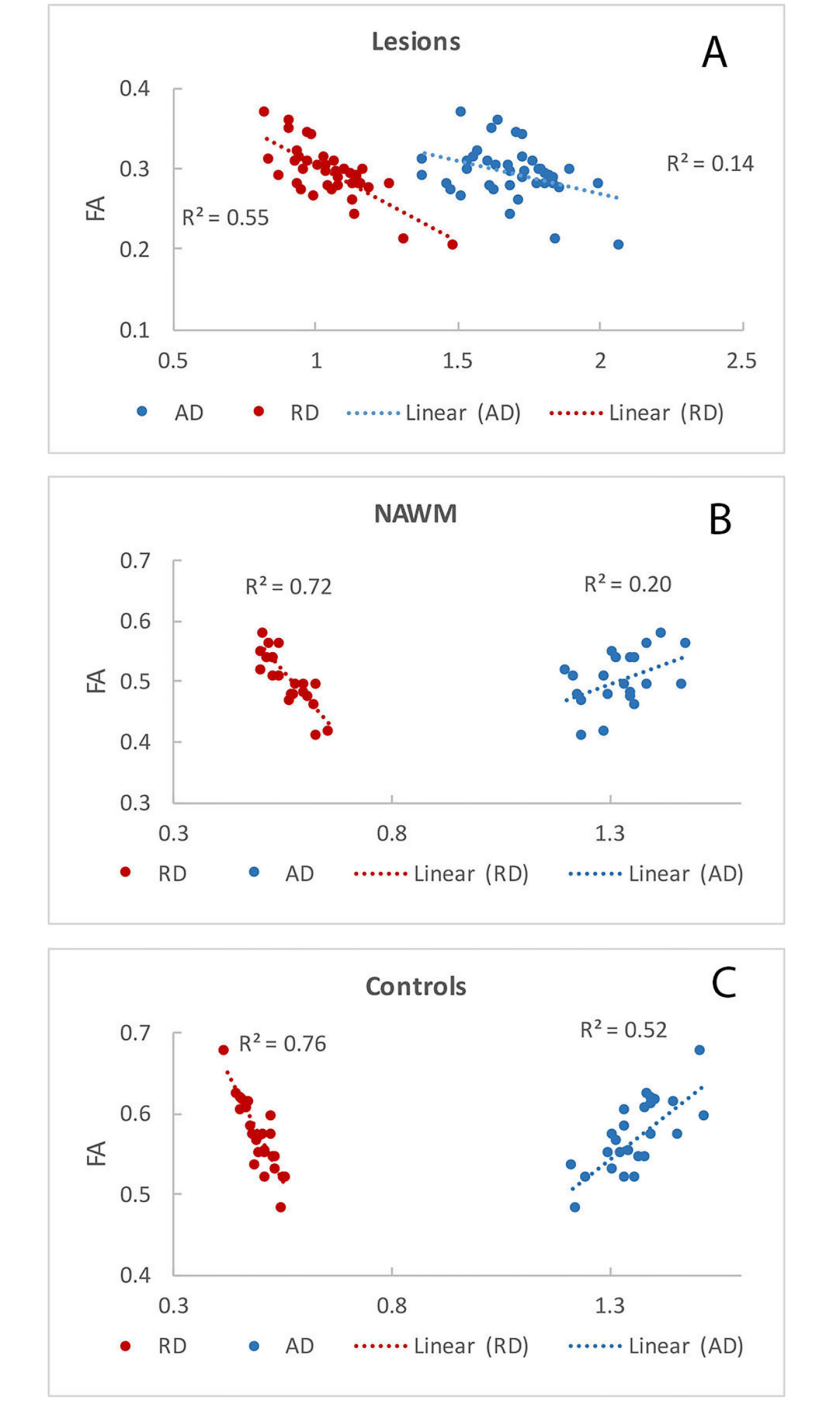
Correlation between anisotropy (FA) and parallel and perpendicular diffusivities (AD and RD) in OR lesions (a), OR NAWM (b) and OR of normal controls (c). Horizontal axes represent diffusivity values x10^−3^ mm^2^ s^−1^.

While the range of parallel and perpendicular diffusivity values in lesional tissue varied considerably, the anisotropy (FA) was predominantly driven by the perpendicular diffusivity (r: λ_1_ = -0.36, λ_2_ = -0.73, λ_3_ = -0.74, RD = -0.74) (Fig 4a). Furthermore, all eigenvalues correlated negatively with FA, meaning that increase of both parallel and perpendicular diffusivities resulted in decline of anisotropy. This relationship is expected for perpendicular diffusivities, but seemed counterintuitive for diffusivity along the fiber orientation (λ_1_).

While anisotropy in NAWM was also mainly driven by perpendicular diffusivity (r: λ_1_ = 0.45, λ_2_ = -0.90, λ_3_ = -0.75, RD = -0.89), contrary to lesional tissue parallel diffusivity demonstrated a positive correlation with FA (Fig 4b). A similar relationship was observed in normal controls, where, however, parallel and perpendicular diffusivity more equitably contributed to the variation in anisotropy (r: λ_1_ = 0.72, λ_2_ = -0.89, λ_3_ = -0.69, RD = -0.87) (Fig 4c).

AD and RD voxel-based histograms in the whole brain lesions demonstrated the highest diffusivity values close to diffusivity of free water (3x10^−3^ mm^2^ s^−1^ and 2.7x10^−3^ mm^2^ s^−1^ for AD and RD respectively). However, the maximum AD and RD values found in OR lesions reached only 2.5 x10^−3^ mm^2^ s^−1^ and 1.7 x10^−3^ mm^2^ s^−1^ respectively, indicating some degree of diffusion restriction and residual anisotropy even in severely damaged white matter. Therefore, both “unrestricted ECS water diffusion” (AD = 3x10^−3^ mm^2^ s^−1^) and “restricted (or rather hindered) ECS water diffusion” (AD = 2.5 x10^−3^ mm^2^ s^−1^) models were used to calculate the proportion of lesional ECS. “Unrestricted ECS water diffusion” model assumes unrestricted and isotropic diffusion of water in lesional extra-cellular space, while “restricted ECS water diffusion” assumes that water diffusion in extra-cellular space of the lesions occupying highly cohesive fiber tracts is hindered and to some degree anisotropic.

The ECS component constituted on average 31.5±13.1% (range 1 to 63%) or 22.0±9.0% (range 1 to 44%) of OR lesional volume for the “unrestricted ECS water diffusion” and “restricted ECS water diffusion” model respectively.

**Fig 5:**
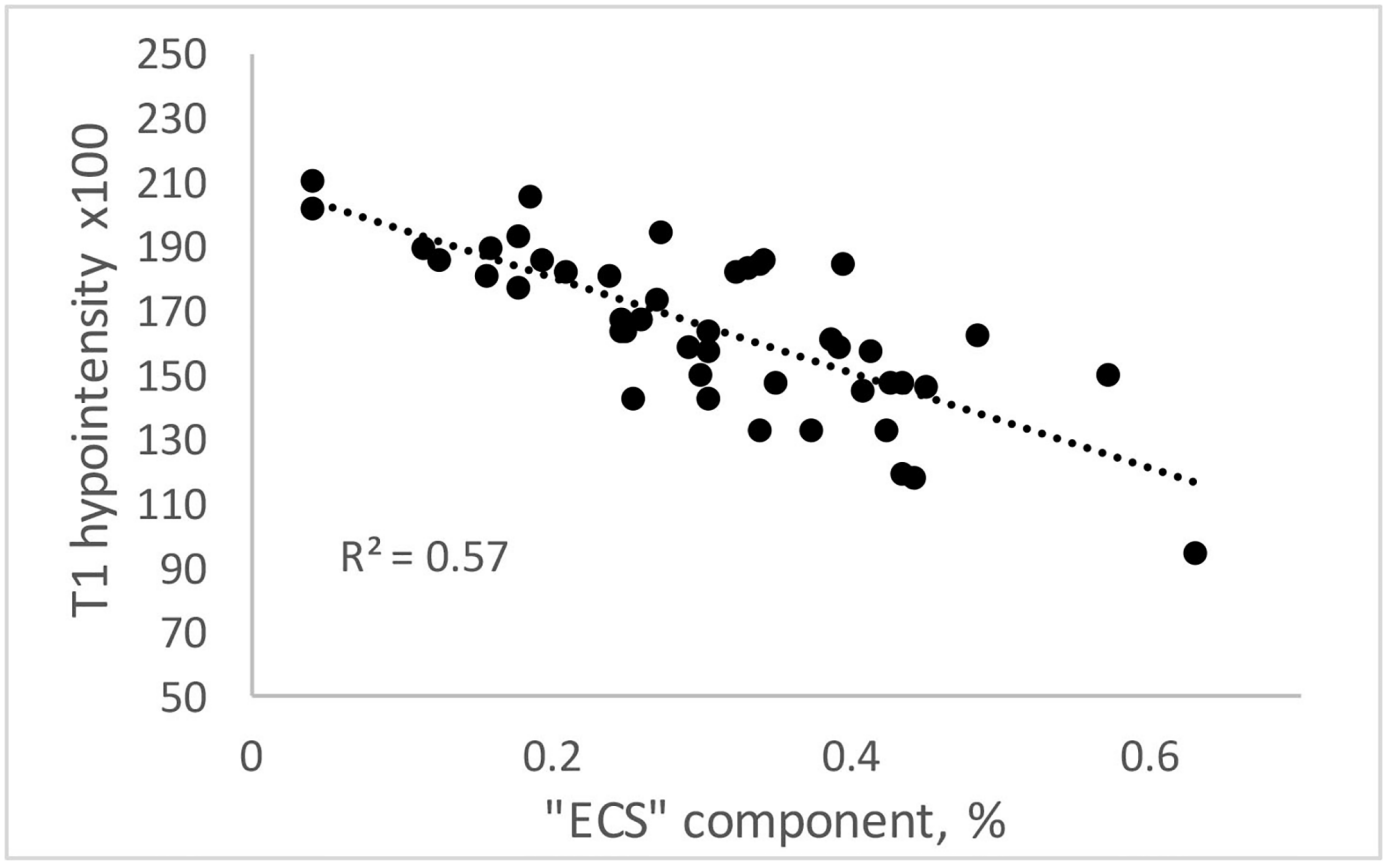
Correlation between proportion of ECS component and T1 hypointensity in optic radiation MS lesions for “restricted ECS water diffusion” model.

There was a highly significant negative correlation between the proportion of ECS component and lesional T1 hypointensity (r = -0.70 and -0.77 for the “unrestricted ECS water diffusion” and “restricted ECS water diffusion” model respectively, p<0.001 for both) (Fig 5).

**Fig 6:**
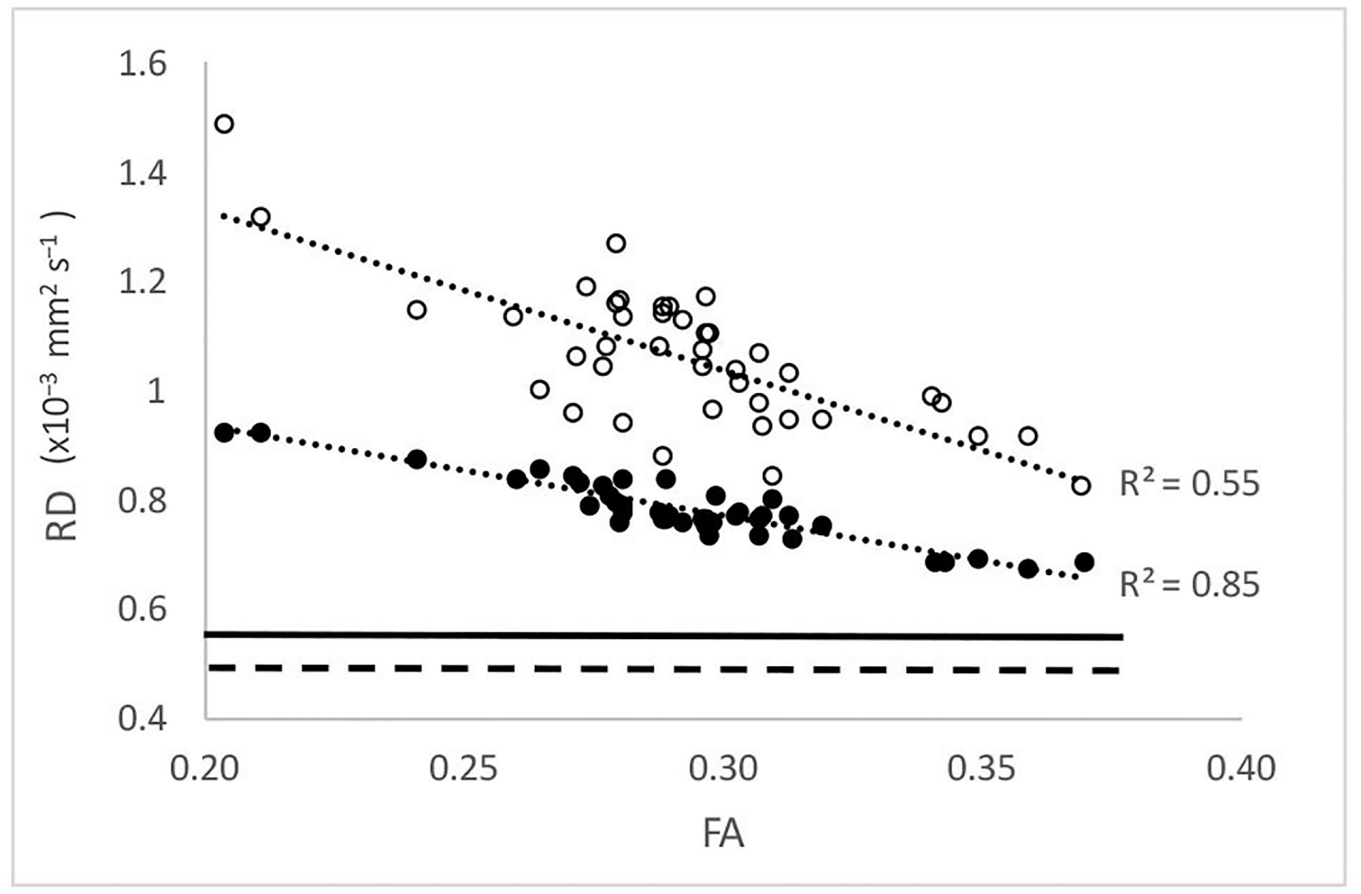
Correlation of observed (empty circles) and normalized (solid circles) RD with fractional anisotropy in MS lesions. The solid horizontal line represents average RD of OR NAWM, the dashed horizontal line average RD of the OR in normal controls.

Based on the estimated proportion of the ECS component, the perpendicular eigenvalues (λ_2_,_3_) were normalised to eliminate the effect of the expanded ECS. As expected, the result of normalisation was independent of the model of ECS water diffusion. It demonstrated that, while values of normalised “residual” perpendicular diffusivity dropped significantly (from 1.17±0.12 to 0.91±0.06 and from 0.97±0.13 to 0.69±0.06 for λ2 and λ3 respectively, or from 1.07±0.13 to 0.8±0.06 for RD), they remained significantly higher compared with perpendicular diffusivity in OR NAWM (p<0.001 for all). In addition, the normalised “residual” perpendicular diffusivity exhibited a very high correlation with the “original” FA, supporting the notion that loss of myelin is the major determinant of reduced anisotropy in the core of chronic MS lesions (Fig 6). This was corroborated by the fact that lesional FA, while increased after re-calculation using “normalised” diffusivity values (from FA = 0.29 to FA = 0.33, p<0.0001 paired t-test), remained considerably lower than FA observed in NAWM (FA = 0.50).

**Fig 7:**
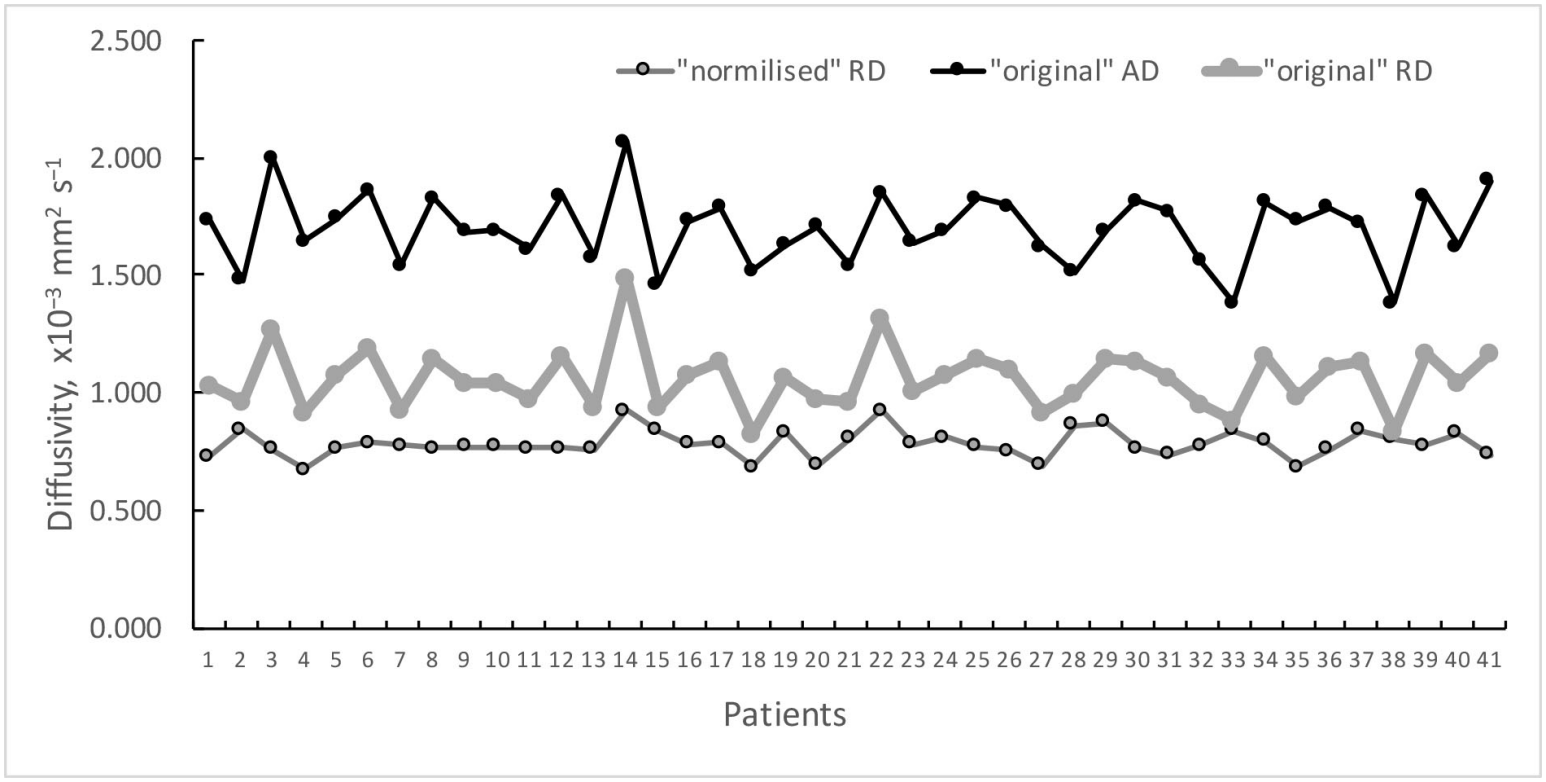
Individual values of observed (“original”) AD and RD diffusivities and “normalised” RD in OR MS lesions.

The “residual” perpendicular diffusivity also showed a substantial reduction of inter-subject variability (i.e. from 11.9% to 7.4% for RD), suggesting more uniform distribution across the patient cohort (Fig 7).

There was a weak correlation between the “residual” perpendicular diffusivity and T1 hypointensity (r = -0.4, p<0.02).

### Simulating diffusivity in the ECS

Based on the hypothesis that increase of AD is largely determined by enlargement of the ECS, we calculated the optimal value of RD in the ECS assuming that complete elimination of the ECS component from RD would minimise the correlation between AD and “residual” RD. Therefore, the formula used above to calculate *f* was modified as follows:

$${RD}^{measured}=f∙{RD}^{residual}+(1-f)∙{RD}^{ECS}$$

or

$${RD}^{residual}=({RD}^{measured}-(1-f)∙{RD}^{ECS})/f$$

**Fig 8:**
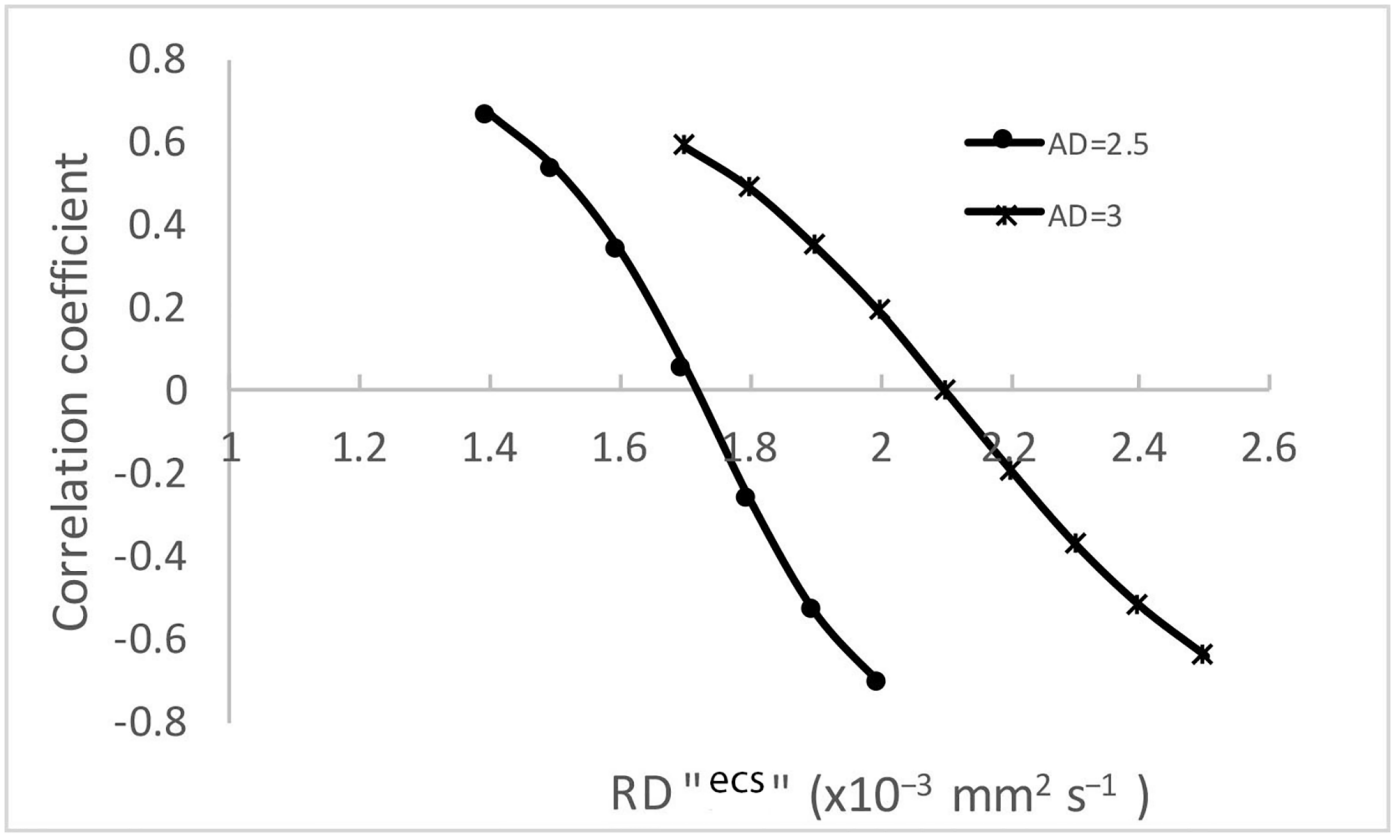
Correlation coefficients between AD and RD^residual^. horizontal axe represents variation in RD^ECS^.

The value of RD^ECS^ was then modulated between 1.5 and 3 in 0.1 steps and correlation between AD and RD^residual^ calculated for each step. This procedure was applied to both “restricted” and “unrestricted” ECS water diffusion models (i.e. *f* calculated based on 2.5 x10^−3^ mm^2^ s^−1^ or 3.0 x10^−3^ mm^2^ s^−1^). For AD value of 2.5 x10^−3^ mm^2^ s^−1^ the correlation dropped to zero at RD^ECS^ = 1.73 x10^−3^ mm^2^ s^−1^ and then re-emerged with the opposite sign, while for AD value of 3 x10^−3^ mm^2^ s^−1^ the minimum correlation was reached at RD^ECS^ = 2.1 x10^−3^ mm^2^ s^−1^ (Fig 8). In both cases the AD^ECS^ /RD^ECS^ ratio was the same (1.4), indicating significant anisotropy of ECS diffusion regardless of the selected AD value. For AD of 2.5 x10^−3^ mm^2^ s^−1^ (the largest observed AD in OR lesions), the value of RD^ECS^ that demonstrated the least correlation with AD (1.73 x10^−3^ mm^2^ s^−1^) was similar to the largest experimentally observed value of RD in OR lesions (1.7 x10^−3^ mm^2^ s^−1^).

Taken together, those findings suggest that the diffusion of water in the ECS of the OR is not only hindered, but is likely to display an anisotropic nature. Therefore, we used anisotropic diffusion of ECS (equal to maximum values found in OR lesions, i.e. AD = 2.5x10^−3^ mm^2^ s^−1^ and RD = 1.7x10^−3^ mm^2^ s^−1^) to simulate the relationship between eigenvalues and FA.

### Eigenvalues and anisotropy

**Fig 9:**
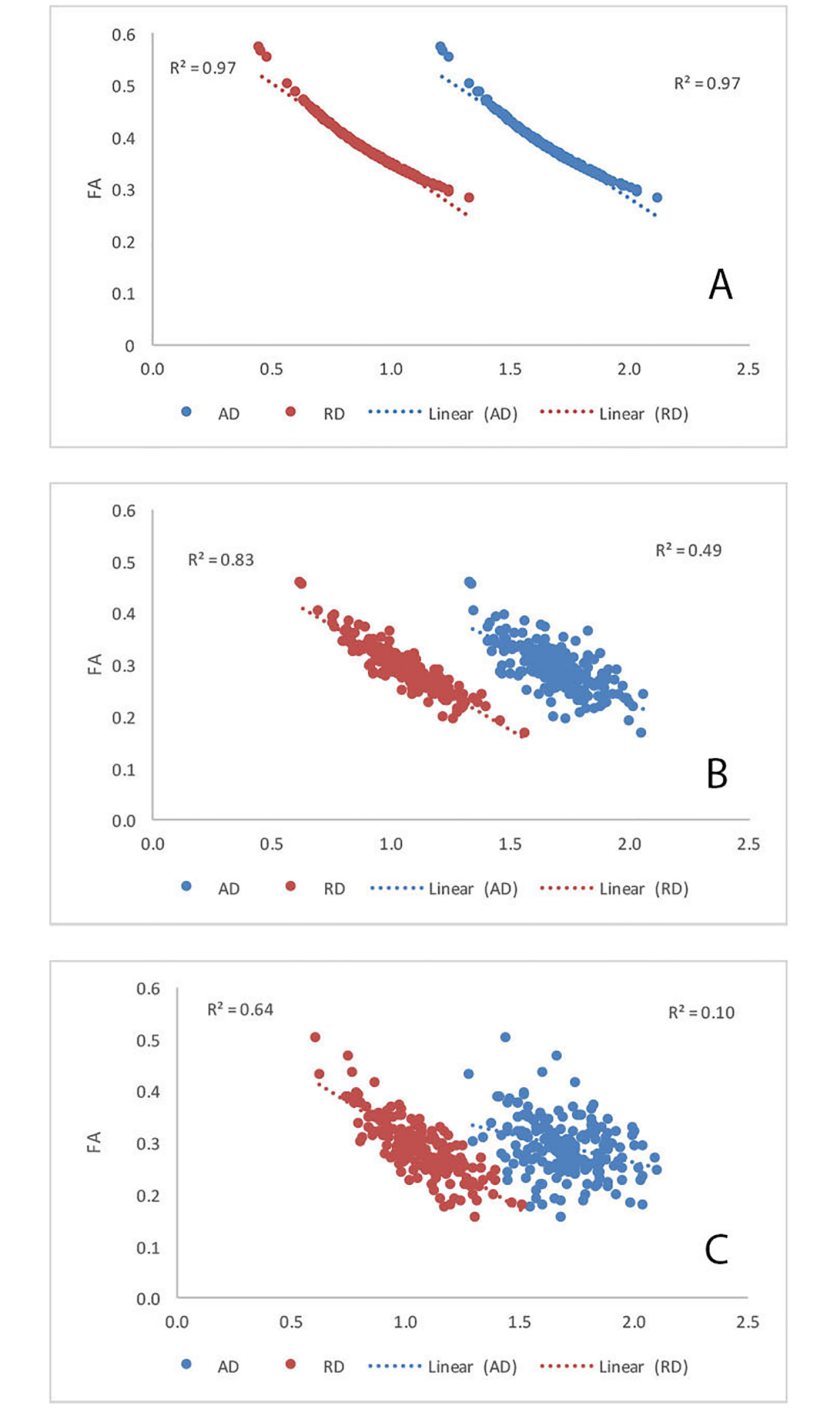
Simulated correlation between anisotropy (FA) and parallel and perpendicular diffusivities (AD and RD): a) stage 1: the expansion of ECS b) stage 2: addition of “membranal” component c) stage 3: addition of inter-subject noise. Horizontal axes represent diffusivity values x10^−3^ mm^2^ s^−1^.

An expansion of ECS results in increase of water diffusion in all directions and, consequently, increase of all eigenvalues, but is likely to produce a reduction of anisotropy caused by a proportionally larger increase of RD (due to its initial low value). We modelled this process by using the first two components of the linear equation describing total eigenvalue in MS lesions (i.e. *f* ∙ *λ*^(*normal tissue*)^ and (1 − *f*)∙*λ*^(*ECS*)^, see Methods). The simulation demonstrated similarly high negative correlations of parallel and perpendicular diffusivities with FA (Fig 9a). The addition of the “membranal” component (*λ*^(*membrane*)^) to the perpendicular eigenvalues significantly reduced the correlation of FA with parallel diffusivity, while preserving the relationship between perpendicular diffusivity and anisotropy (Fig 9b). Finally, introducing inter-subject noise into the model (adding respective λ^(noise)^ to all eigenvalues) further reduced correlation coefficients between parallel and perpendicular diffusivity and FA, making them similar to the observed data (r = -0.33±0.4 and -0.81±0.3 for AD and RD respectively, averaged of 50 iterations) (Fig 9c).

## Discussion

The purpose of this study was to isolate the contribution of extra-cellular water and demyelination to observed DTI indices in the core of chronic MS lesions, using the OR as an anatomically cohesive tract. We examined DTI parameters in a cohort of 75 RMMS patients and performed modelling of the results based on an assumption that, in highly cohesive white matter tracts an increase of parallel diffusivity is largely related to the expansion of the ECS caused by tissue destruction, while an increase of perpendicular diffusivity is likely to represent a combined effect of enlarged ECS and membranal (mostly myelin) loss.

There have been numerous attempts to disentangle the pathological processes that lead to changes in diffusivity in brain white matter. While earlier DTI models assumed a 3D Gaussian distribution of the random displacements of water molecules, more advanced methods assume that diffusion within the white matter tissue is a multi-compartmental process involving a combination of Gaussian and non- Gaussian diffusion [19].

In the composite hindered and restricted model of diffusion (CHARMED), the intra-axonal space is represented as a region of restricted diffusion, while water molecules in the extra-axonal space are assigned a hindered mode of diffusion displaying anisotropic properties [19]. Conversely, the bi-exponential model developed by Pierpaoli and Jones, and later modified by Pasternak et al, treated increased volume of ECS as an isotropic tensor with diffusivity of free water[20][21]. A similar approach is used in diffusion basis spectrum imaging (DBSI), a recently proposed technique employing multiple-tensor modelling to discriminate specific pathological components in white matter of MS patients [22] where axons are modeled as an anisotropic diffusion tensor, while the ECS is designated as isotropic diffusion [23]. Diffusion based on a three-compartment models have recently been developed by Barazany et al [24], while as many as four compartments were suggested by Alexander et al[25].

While complex models may help to better explain the observed experimental or clinical data [26], they require very high quality measurements, such as multi-shell high-angular-resolution acquisition or NMR spectroscopy. This may be necessary to estimate axonal diameter distribution and other high-order information, but is not feasible in a clinical setting.

In the current study, we hypothesized that an increase of diffusivity parallel to fiber orientation in highly coherent white matter tracts, such as the OR, is largely caused by the replacement of axonal structure with ECS. Similar to the two-tensor model (which was developed to describe partial volume effect and free water elimination[27][20][21]), we defined voxel-based diffusivity along each eigenvector as a linear combination of diffusivities in two tissue compartments (axonal and extra-axonal) with different diffusion properties (restricted and hindered). However, contrary to previous studies, we estimated the ECS based on AD alone. The rationale for our hypothesis is built on current knowledge of the microstructure of chronic MS lesions, which, in highly coherent tracts, is represented by similarly oriented survived, but denuded, axons, expanded ECS and glial proliferation[28][29]. Conversely, while some changes have been demonstrated in NAWM, it is assumed that its tightly packed structure of myelinated axons is largely intact in OR. While both membranal loss and enlargement of the ECS within lesions are expected to affect diffusion of water molecules in a direction perpendicular to fiber orientation, membranal loss is unlikely to play a significant role in alteration of the diffusivity parallel to axons. Therefore, increased parallel diffusivity relative to NAWM may provide an estimation of the proportion of the expanded ECS (and, consequently, the loss of axonal tissue) within MS lesions. In addition, since the effect of expansion of the ECS on diffusivity is likely to be similar (or at least proportional) in all directions (as supported by a similar relationship between anisotropy and both parallel and perpendicular diffusivities in MS lesions and a close correlation between AD and RD in MS lesions, but not in NAWM), perpendicular eigenvalues can be normalised by the change of AD to eliminate (or minimize) the contribution of expanded ECS. The “residual” (compared to NAWM) perpendicular diffusivity may, therefore, provide a measure of membranal loss, likely reflecting the degree of demyelination.

The current analysis, which was performed on a sizable cohort of MS patients, revealed a wide variance in lesional AD, suggesting (based on the presented hypothesis) varying degrees of ECS enlargement. Increases in AD (relative to NAWM) demonstrated strong correlation with lesional T1 hypointensity, an imaging metric that is closely associated with the degree of tissue destruction within MS lesions[8][30]. This is consistent with previous DTI studies (see [31] for review) and, therefore, support the use of the relative increase of AD as a marker of tissue damage.

The magnitude of tissue destruction identified by our technique, however, is determined by the value assigned to the diffusivity of water in the ECS (i.e. the higher the assumed diffusion in the ECS, the lesser the volume required to reach the observed AD value). Since the true value of ECS diffusion in white matter of human brain is unknown, we examined the voxel-based histogram of AD/RD distribution in the core of OR lesions assuming that, at least in some voxels, the tissue damage is severe enough to cause complete destruction of axonal structure. The maximal AD and RD values, however, never exceeded 2.5x10^−3^ mm^2^ s^−1^ and 1.7x10^−3^ mm^2^ s^−1^ respectively. This indicates that, firstly, the diffusivity in white matter ECS is unlikely to reach the level of free water diffusion (which is equal to 3x10^−3^ mm^2^ s^−1^) and secondly, even in ECS the diffusion remains anisotropic, at least in the OR. Anisotropic diffusion within ECS was also supported by the modeling. Thus, based on correlation between AD and RD, minimization of the ECS contribution to perpendicular diffusivity demonstrated anisotropic diffusion within ECS of similar magnitude to experimentally observed values.

The anisotropic nature of diffusivity in ECS has recently been advocated by several diffusivity models as providing better fit of the experimental data. It was suggested to be related to excessive gliosis and axonal tortuosity [26] [19][32]. The role of glial cells was supported by recent experimental studies demonstrating that fibrous astrocytes, which are primarily responsible for scar formation in white matter, have intrinsically longer processes that are primarily arranged parallel to white matter fibres in the normal brain and in case of injury can give rise to directional cohesiveness [33][18]. However, “axonal” component may also be involved in maintaining anisotropy of the extra-axonal space. Since a relatively small proportion of axons are transected in acute MS lesions, perpendicular diffusivity in the ECS of highly coherent fiber tracts may remain hindered due to increased axonal tortuosity and reflections from axons or neurofilaments and proteins oriented parallel to them [19][32]. The difference between ECS diffusivity in OR and whole brain (which demonstrated the diffusivity values close to diffusivity of free water) is likely to be related to highly coherent nature of OR fibers, while crossing fibers in other brain regions may obscure this phenomenon, resulting in more similar diffusivity in all directions.

While the selected AD value for the ECS did affect calculation of the degree of tissue destruction, it had no impact on proportional scaling of the perpendicular diffusivity, which demonstrated a significant residual value compared to the diffusivity of OR NAWM. This “residual” diffusivity is likely to be related to the loss of structures perpendicular to the fiber orientation. While there are two potential candidates for this role, namely axonal membranes and myelin sheath, we believe that it is the loss of myelin that is largely responsible for this phenomenon, since disintegration of axonal membrane, which ultimately leads to axonal death and subsequent replacement with ECS, is already accounted for by the increase of ECS. Considerably smaller inter-subject variability also supports demyelination as a major pathophysiological mechanism underlying the “residual “perpendicular diffusivity since largely complete and similar demyelination is expected in the core of chronic lesions in MS patients.

Despite the lesser variability, the normalised “residual” perpendicular diffusivity exhibited a very high correlation with “original” FA, supporting the notion that in MS lesions the loss of myelin membranes plays the major role in reduction of anisotropy. This relationship became particularly apparent after the component attributed to increase of the ECS was removed. The contribution of membranal loss to anisotropy is also reinforced by the fact that FA recalculated based on “normalised” values of diffusivity remained considerably lower compared to anisotropy observed in NAWM.

It is also worth noting that diffusion anisotropy in MS lesions was largely driven by the perpendicular diffusivity. The strength of the (negative) correlation between FA and RD increased even further after a normalization procedure was applied to remove the effect of ECS. Conversely, there was a weak negative correlation between FA and AD, which was rather surprising considering that higher AD values in white matter are expected to produce a higher FA (as was found in NAWM and normal controls).

While the negative nature of the correlation between FA and AD in MS lesions can be explained by a proportionally larger increase of RD in response to expansion of the ECS (due to its lower value in normal tissue), its low strength is probably related to the presence of a “residual” demyelinating component of RD and larger inter-subject variability of AD compared to perpendicular diffusivity (both of those factors represent an additional “noise” in relation to AD, reducing its effect on anisotropy). This is clearly seen following the implementation of consecutive stages of the simulation. Thus, the expansion of ECS (stage 1) demonstrated high (and identical) negative correlations of all eigenvalues with FA. Addition of the “residual” demyelinating component (stage 2) resulted in a significant fall in the correlation between FA and AD, while correlation with RD remained largely unaltered. Finally, adding the inter-subject variability (stage 3) further reduced the correlation for both AD and RD yielding values similar to the observed data.

There are several potential limitations in this study. In particular, the current assumption that ECS can be estimated based on AD alone is only valid in highly coherent tracts, such as optic radiation, where effect of crossing fibers in negligible. Furthermore, we have assumed a Gaussian diffusion model. However, using a highly coherent pathway such as the OR significantly limits the potential negative effect of variation in axonal orientation (i.e. crossing, kissing, bending or fanning), a major limitation of DTI techniques[34] [17]. When applied to well-defined tightly packed white matter tracts without inflammation (as is the case in the current study), DTI is likely to accurately reflect the status of tissue water diffusion[35] [36]. In addition, we believe that using the lesion “core”, as opposed to the entire lesion, not only removes uncertainty related to possible “slow burning”inflammation and de/remyelination at the lesion edge, but also helps to eliminate the partial volume effect from CSF (since OR lesions are located in close proximity to the lateral ventricles). No water exchange between compartments was assumed, which also may be an oversimplification.

In addition, myelin constitutes a significant proportion of normal white matter volume. While diffusion of water within the myelin sheaths is negligible due to its very short relaxation time[37], what happens to the space vacated by demyelination (and how it may affect parallel diffusivity) is not known. This space may simply collapse, be filled by astrocytes or replaced by extra-cellular matrix or a combination of these. This may potentially have different consequences for diffusivity, which was not considered in the current study. We believe, however, that its potential effect on diffusivity is minimal since, while in the lesion “core” the degree of demyelination (and, therefore, space vacated by myelin) is likely to be similar between patients, some of the patients demonstrated normal or near normal parallel diffusivity, indicating little interaction between loss of myelin and AD. A minimal contribution of demyelination to the expansion of ECS is also supported by the weak correlation found between “residual” perpendicular diffusivity and T1 hypointensity.

Relatively low resolution of DTI (compare to structural imaging) may also be a potential confounding factor in this study.

In conclusion, DTI provides a unique *in vivo* insight into the dynamic nature of MS pathology. Understanding the pathological mechanisms responsible for altered diffusivity in MS will critically determine the utility of DTI in future clinical trials. Single tract-based approaches are increasingly popular due to relatively coherent axonal structure, which reduces acquisition requirements and considerably simplifies data interpretation. The current paper presents a potential technique for disentangling and quantifying the effects of neurodegeneration (tissue loss) and demyelination in OR MS lesions. This was achieved by identifying the diffusivity component relating to increased ECS and removing its effect on perpendicular diffusivity. This technique may provide a simple and effective means for applying single tract diffusion analysis in MS clinical trials, with particular relevance to pro-remyelinating and neuroprotective therapeutics.

## Supporting information

S1 File Minimal data set Plos one.xlsx. Minimal data set.
(XLSX)

## References

[1] Compston A, Coles A. Multiple sclerosis. *Lancet* 2008;372:1502–11. doi: 10.1016/S0140-6736(08)61620-7 18970977 10.1016/S0140-6736(08)61620-7

[2] Schmierer K, Wheeler-Kingshott CAM, Boulby PA, Scaravilli F, Altmann DR, Barker G, et al. Diffusion tensor imaging of post mortem multiple sclerosis brain. *Neuroimage* 2007;35:467–77. doi: 10.1016/j.neuroimage.2006.12.010 17258908 10.1016/j.neuroimage.2006.12.010 PMC1892244

[3] Walhovd KB, Ka RT. Unraveling the secrets of white matter. *Neurosci* 2014;276:2–13. 25003711 10.1016/j.neuroscience.2014.06.058 PMC4155933

[4] Jones DK, Knösche TR, Turner R. White matter integrity, fiber count, and other fallacies: The do ‘ s and don ‘ ts of diffusion MRI. *Neuroimage* 2013;73:239–54. doi: 10.1016/j.neuroimage.2012.06.081 22846632 10.1016/j.neuroimage.2012.06.081

[5] Barnes D, Munro PM, Youl BD, Prineas JW, McDonald WI. The longstanding MS lesion. A quantitative MRI and electron microscopic study. *Brain* 1991;114:1013–23. 2065249 10.1093/brain/114.3.1271

[6] Miller DH. Neuroimaging in multiple sclerosis In: Raine CS, Mcfarland HF, Hohlfeld R, eds. *Multiple sclerosis*. Edinburgh:: Elsevier 2008 69–87.

[7] Werring DJ, Clark CA, Barker GJ, Thompson AJ, Miller DH. Diffusion tensor imaging of lesions and normal-appearing white matter in multiple sclerosis. *Neurology* 1999;52:1626–1626. doi: 10.1212/WNL.52.8.1626 10331689 10.1212/wnl.52.8.1626

[8] van Walderveen MA, Kamphorst W, Scheltens P, van Waesberghe JH, Ravid R, Valk J, et al. Histopathologic correlate of hypointense lesions on Tl-weighted spin-echo lesions in multiple sclerosis MRI. *Neurology* 1998;50:1282–8. 9595975 10.1212/wnl.50.5.1282

[9] Bammer R, Augustin M, Strasser-Fuchs S, Seifert T, Kapeller P, Stollberger R, et al. Magnetic resonance diffusion tensor imaging for characterizing diffuse and focal white matter abnormalities in multiple sclerosis. *Magn Reson Med* 2000;44:583–91. doi: 10.1002/1522-2594(200010)44:4<583::AID-MRM12>3.0.CO;2-O 11025514 10.1002/1522-2594(200010)44:4<583::aid-mrm12>3.0.co;2-o

[10] Mädler B, Drabycz S a, Kolind SH, Whittall KP, MacKay AL. Is diffusion anisotropy an accurate monitor of myelination? Correlation of multicomponent T2 relaxation and diffusion tensor anisotropy in human brain. *Magn Reson Imaging* 2008;26:874–88. doi: 10.1016/j.mri.2008.01.047 18524521 10.1016/j.mri.2008.01.047

[11] Yeatman JD, Dougherty RF, Myall NJ, Wandell BA, Feldman HM. Tract profiles of white matter properties: automating fiber-tract quantification. *PLoS One* 2012;7:e49790 doi: 10.1371/journal.pone.0049790 23166771 10.1371/journal.pone.0049790 PMC3498174

[12] Winston GP. The physical and biological basis of quantitative parameters derived from diffusion MRI. *Quant Imaging Med Surg* 2012;2:254–65. doi: 10.3978/j.issn.2223-4292.2012.12.05 23289085 10.3978/j.issn.2223-4292.2012.12.05 PMC3533595

[13] Wheeler-Kingshott CAM, Cercignani M. About ‘Axial’ and ‘Radial’ Diffusivities. *Mag Res Med* 2009;61:1255–60. 10.1002/mrm.21965 19253405

[14] Polman CH, Reingold SC, Banwell B, Clanet M, Cohen JA, Filippi M, et al. Diagnostic criteria for multiple sclerosis: 2010 revisions to the McDonald criteria. *Ann Neurol* 2011;69:292–302. doi: 10.1002/ana.22366 21387374 10.1002/ana.22366 PMC3084507

[15] Klistorner A, Vootakuru N, Wang C, Yiannikas C, Graham SL, Parratt J, et al. Decoding diffusivity in multiple sclerosis: analysis of optic radiation lesional and non-lesional white matter. *PLoS One* 2015;10:e0122114 doi: 10.1371/journal.pone.0122114 25807541 10.1371/journal.pone.0122114 PMC4373765

[16] Sherbondy AJ, Dougherty RF, Napel S, Wandell BA. Identifying the human optic radiation using diffusion imaging and fiber tractography. *J Vis* 2008;8:1–11. 10.1167/8.10.12 PMC2759943 19146354

[17] Vos SB, Jones DK, Viergever MA, Leemans A. Partial volume effect as a hidden covariate in DTI analyses. *Neuroimage* 2011;55:1566–76. doi: 10.1016/j.neuroimage.2011.01.048 21262366 10.1016/j.neuroimage.2011.01.048

[18] Budde MD, Janes L, Gold E, Turtzo LC, Frank JA. The contribution of gliosis to diffusion tensor anisotropy and tractography following traumatic brain injury: validation in the rat using Fourier analysis of stained tissue sections. *Brain* 2011;134:2248–60. doi: 10.1093/brain/awr161 21764818 10.1093/brain/awr161 PMC3155707

[19] Assaf Y, Freidlin RZ, Rohde GK, Basser PJ. New Modeling and Experimental Framework to Characterize Hindered and Restricted Water Diffusion in brain white matter. *Magn Reson Med* 2004;52:965–78. doi: 10.1002/mrm.20274 15508168 10.1002/mrm.20274

[20] Pierpaoli C, Jones DK. Removing CSF Contamination in Brain DT-MRIs by Using a Two-Compartment Tensor Model. Proc Int Soc Magn Reson Med 2004;11:1215.

[21] Pasternak O, Sochen N, Gur Y, Intrator N, Assaf Y. Free water elimination and mapping from diffusion MRI. *Magn Reson Med* 2009;62:717–30. doi: 10.1002/mrm.22055 19623619 10.1002/mrm.22055

[22] Wang Y, Sun P, Wang Q, Trinkaus K, Schmidt RE, Naismith RT, et al. Differentiation and quantification of inflammation, demyelination and axon injury or loss in multiple sclerosis. *Brain* 2015;138:1223–38. doi: 10.1093/brain/awv046 25724201 10.1093/brain/awv046 PMC4407189

[23] Chiang C-W, Wang Y, Sun P, Lin TH, Trinkaus K, Cross AH, et al. Quantifying white matter tract diffusion parameters in the presence of increased extra-fiber cellularity and vasogenic edema. *Neuroimage* 2014;101:310–9. doi: 10.1016/j.neuroimage.2014.06.064 25017446 10.1016/j.neuroimage.2014.06.064 PMC4165711

[24] Barazany D, Basser PJ, Assaf Y. In vivo measurement of axon diameter distribution in the corpus callosum of rat brain. *Brain* 2009;132:1210–20. doi: 10.1093/brain/awp042 19403788 10.1093/brain/awp042 PMC2677796

[25] Alexander DC, Hubbard PL, Hall MG, Moore EA, Ptito M, Parker GJ, et al. Orientationally invariant indices of axon diameter and density from diffusion MRI. *Neuroimage* 2010;52:1374–89. doi: 10.1016/j.neuroimage.2010.05.043 20580932 10.1016/j.neuroimage.2010.05.043

[26] Panagiotaki E, Schneider T, Siow B, Hall MG, Lythgoe MF, Alexander DC. Compartment models of the diffusion MR signal in brain white matter : A taxonomy and comparison. *Neuroimage* 2012;59:2241–54. doi: 10.1016/j.neuroimage.2011.09.081 22001791 10.1016/j.neuroimage.2011.09.081

[27] Alexander AL, Hasan KM, Lazar M, Tsuruda JS, Parker DL. Analysis of Partial Volume Effects in Diffusion-Tensor MRI. *Mag Res Med* 2001;45:770–80. 10.1002/mrm.1105 11323803

[28] Barnes D, Munro PM, Youl BD, Prineas JW, McDonald WI. The longstanding MS lesion. A quantitative MRI and electron microscopic study. *Brain* 1991;114 (Pt 3:1271–80. http://eutils.ncbi.nlm.nih.gov/entrez/eutils/elink.fcgi?dbfrom=pubmed&id=2065249&retmode=ref&cmd=prlinks\npapers2://publication/uuid/0D6BB3C9-79EF-4AA9-9900-40D5E420A833 2065249 10.1093/brain/114.3.1271

[29] Ormerod IE, Miller DH, McDonald WI, du Boulay EP, Rudge P, Kendall BE, et al. The role of NMR imaging in the assessment of multiple sclerosis and isolated neurological lesions. A quantitative study. *Brain* 1987;110 (Pt 6:1579–616. http://www.ncbi.nlm.nih.gov/pubmed/3427402 3427402 10.1093/brain/110.6.1579

[30] van Waesberghe JH, Kamphorst W, De Groot CJ, van Walderveen MA, Castelijns JA, Ravid R, et al. Axonal loss in multiple sclerosis lesions: magnetic resonance imaging insights into substrates of disability. *Ann Neurol* 1999;46:747–54.http://www.ncbi.nlm.nih.gov/pubmed/10553992 10553992 10.1002/1531-8249(199911)46:5<747::aid-ana10>3.3.co;2-w

[31] Sbardella E, Tona F, Petsas N, Pantano P. DTI Measurements in Multiple Sclerosis: Evaluation of Brain Damage and Clinical Implications. *Mult Scler Int* 2013;2013:671730 doi: 10.1155/2013/671730 23606965 10.1155/2013/671730 PMC3628664

[32] Zhang H, Schneider T, Wheeler-kingshott CA, Alexander DC. NODDI : practical in vivo neurite orientation dispersion and density imaging of the human brain. Neuroimage 2012; 61:1000–16 doi: 10.1016/j.neuroimage.2012.03.072 22484410 10.1016/j.neuroimage.2012.03.072

[33] Bitner C, Benjelloun-Touimi S, Dupouey P. Palisading pattern of subpial astroglial processes in the adult rodent brain: relationship between the glial palisading pattern and the axonal and astroglial organization. *Brain Res* 1987;15:167–78. 10.1016/0165-3806(87)90238-0 3440199

[34] Descoteaux M. HIGH ANGULAR RESOLUTION DIFFUSION IMAGING (HARDI) In: Wiley J, ed. *The Wiley Encyclopedia of Electrical and Electronics Engineering*. John Wiley & Sons, Inc 2015 1–25. doi: 10.1002/047134608X.W8258

[35] Naismith RT, Xu J, Tutlam NT, Scully PT, Trinkaus K, Snyder AZ, et al. Increased diffusivity in acute multiple sclerosis lesions predicts risk of black hole. *Neurology* 2010;74:1694–701. doi: 10.1212/WNL.0b013e3181e042c4 20498437 10.1212/WNL.0b013e3181e042c4 PMC2882210

[36] Oh J, Saidha S, Chen M, Smith SA, Prince J, Jones C, et al. Spinal cord quantitative MRI discriminates between disability levels in multiple sclerosis. *Neurology* 2013;80:540–7. doi: 10.1212/WNL.0b013e31828154c5 23325903 10.1212/WNL.0b013e31828154c5 PMC3589285

[37] Beaulieu C. The basis of anisotropic water diffusion in the nervous system—a technical review. *NMR Biomed* 2002;15:435–55. doi: 10.1002/nbm.782 12489094 10.1002/nbm.782

